# The Interaction between Education and Sex with Alcohol Consumption during the COVID-19 Pandemic: A Cross-Sectional Analysis of Two Brazilian Cities

**DOI:** 10.3390/ijerph21060804

**Published:** 2024-06-19

**Authors:** Amanda Popolino Diniz, Raquel de Deus Mendonça, George Luiz Lins Machado-Coelho, Adriana Lúcia Meireles

**Affiliations:** 1 Postgraduate Program in Health and Nutrition, Nutrition School, Federal University of Ouro Preto, Ouro Preto 35400000, MG, Brazil; amanda.diniz@aluno.ufop.br; 2 Department of Clinical and Social Nutrition, School of Nutrition, Federal University of Ouro Preto, Ouro Preto 35400000, MG, Brazil; raquel.mendonca@ufop.edu.br; 3 Epidemiology Laboratory, Medical School, Federal University of Ouro Preto, Ouro Preto 35400000, MG, Brazil; gmcoelho@ufop.edu.br

**Keywords:** binge drinking, COVID-19, alcohol, socioeconomic level, cross-sectional studies

## Abstract

This cross-sectional study, carried out between October and December 2020 in two Brazilian cities, aimed to evaluate the joint association of education and sex with habitual and episodic excessive alcohol consumption during the COVID-19 pandemic. Habitual alcohol consumption was defined as drinking any quantity of alcohol at least once per week. Excessive episodic alcohol consumption was defined as the consumption of five or more drinks by men or four or more drinks by women at least once in the last 30 days. Adjusted multivariate logistic regression models were used to analyze associations of education and sex with alcohol consumption. Education was not associated with habitual alcohol consumption and excessive episodic alcohol consumption. However, when evaluating the joint effect between education and sex, it can be seen that men with low education were more likely to habitually consume (OR: 5.85; CI95:2.74–14.84) and abuse alcohol (OR: 4.45; IC95:1.54–12.82) and women with high education were more likely to have habitual (OR: 2.16; IC95:1.18–3.95) and abusive alcohol consumption (OR: 2.00; IC95:1.16–3.43). These findings highlight the modifying effect of sex on the relationship between education and alcohol consumption, such that education influenced alcohol consumption differently between sexes during the pandemic.

## 1. Introduction

Alcohol consumption is a risk factor for morbidity and mortality, both in Brazil and worldwide. According to the Global Status Report on Alcohol and Health published by the World Health Organization in 2018, alcohol consumption is responsible for more than 3 million deaths every year and more than 5% of the total burden of disease and injuries around the world [1].

The consumption of alcoholic beverages varies among the Brazilian population according to factors such as age, ethnicity, skin color, socioeconomic status, education, work, marital status, and neighborhood characteristics, and these factors may differ between men and women [2,3,4,5].

Men are more likely to drink than women, and differences in consumption refer to the amount and way they drink, in addition to the social and health aspects to which they are subject [2,6]. The frequency of consumption of once or more per week by the Brazilian population in 2019 was 26.4% (37.1% for men; 17.0% for women) [7].

However, excessive episodic alcohol consumption has increased, particularly among women [1,6]. In Brazil, the frequency of abusive consumption of alcoholic beverages has increased from 15.7% in 2006 to 20.9% in 2020 [8]. This growth trend is greater among women, ranging from 7.8% in 2006 to 16.0% in 2020 [8]. Among males, excessive episodic alcohol consumption ranged from 25.0% in 2006 to 26.6% in 2020 [8]. The convergence of prevalence according to sex has been described in the literature [9,10,11]; however, few studies have investigated this phenomenon in Brazil, especially in the context of a health crisis such as the COVID-19 pandemic.

Furthermore, epidemiological studies have shown divergent associations between education level and alcohol consumption. Some studies have demonstrated an association between high education and/or income and excessive episodic alcohol consumption [3,12], whereas others have revealed a higher proportion of drinkers among individuals with less education [5,13,14,15] or found no association at all [4]. These findings reflect the complex relationship between education and alcohol consumption patterns, highlighting the need for further investigation.

Although numerous studies have analyzed alcohol consumption according to sociodemographic factors, most have evaluated these factors in isolation and/or stratified by sex [3,4,5]. In addition, the COVID-19 pandemic has influenced alcohol consumption in the general population, especially in socioeconomically vulnerable groups [16,17]. Studies carried out during the pandemic indicated an increase in alcohol consumption in some countries [18,19,20]. Data from the Pan American Health Organization’s regional survey demonstrated that 65% of individuals had excessive episodic alcohol consumption and that men were more likely to practice this pattern of alcohol consumption during the pandemic [6].

The scenario experienced by the reduction in social interactions and changes in routine and financial conditions predisposed the population to anxiogenic and stressful situations that may have affected health behaviors, highlighting a negative effect on alcohol consumption [21,22]. Furthermore, the multifaceted nature of the impact of the pandemic in the context of the pre-existing social and economic fragility in Brazil [23] suggests analyzing the consumption of alcoholic beverages from the perspective of the population’s socioeconomic conditions. Therefore, the hypothesis of this study was that, as a result of social restriction measures, education, together with sex, would worsen the negative scenario for the consumption of this legal psychoactive substance.

Thus, the need to investigate whether the interaction between education and sex is associated with the consumption of alcoholic beverages during the COVID-19 pandemic is reinforced to contribute to the advancement of understanding of a socially complex phenomenon and provide information that improves the effectiveness of health approaches to reducing alcohol consumption during epidemics. Therefore, this study aimed to evaluate the joint association of education and sex with habitual consumption of alcoholic beverages and excessive episodic alcohol consumption during the COVID-19 pandemic.

## 2. Materials and Methods

### 2.1. Study Design and Population

This is a cross-sectional study carried out through a population-based household seroepidemiological survey, “Epidemiological surveillance of COVID-19 in the Inconfidentes Region/MG (COVID-Inconfidentes Project)”, carried out between October and December 2020.

The study was conducted in Ouro Preto, with a municipal human development index of 0.741, and Mariana, with a municipal human development index of 0.742. These are considered medium-sized cities in the central-south region of Minas Gerais, known as Quadrilátero Ferrífero, which is one of the largest iron ore-producing areas in Brazil. According to the 2010 demographic census, the municipality of Ouro Preto had a total population of 70,281 inhabitants, and Mariana had a total of 54,219 inhabitants living in the urban area, distributed in 17,753 and 14,078 households in Ouro Preto and Mariana, respectively [24].

A sampling design was adopted using probabilistic sampling stratified by a conglomerate in three stages: census, household, and residential. This design was based on large national household surveys, such as the National Household Sample Survey [25], the Family Budget Survey [26], the Health in Beagá Survey [27], and, more recently, the EPICOVID19 study [28].

The sample calculation was performed using the OpenEpi tool based on the 2010 population estimate [24] for each city, adopting a confidence level of 95% and an effect size of 1.5. In addition, 20% was added to the sample size of each city for possible refusals, absence of selected residents, or people who were not at home during the visit. Participants eligible for the study were permanent residents of households in the urban areas of the municipalities, aged ≥ 18 years, who consented to participate in the study. Individuals with compromised cognitive function, difficulty in responding to the questionnaire, or inability to provide blood samples due to difficulties with venous access were excluded [29].

The sampling weight of each selected unit (census sector, household, and individual) was calculated to correlate with the 2019 population projection. For more details on the sample calculation and field logistics, see Meireles et al. [29]. A total of 1789 individuals were recruited, of which 27 (1.5%) were excluded for not completing the interview or not collecting a blood sample, totaling 1762 individuals, of which 764 were from the municipality of Mariana (43.4%) and 998 from Ouro Preto (56.6%). Of this total, nine individuals were excluded for not answering the question regarding alcohol consumption, totaling a final sample of 1753 individuals.

### 2.2. Data Collection

In each municipality, data were collected on three weekends (Friday, Saturday, and Sunday) with an interval of 21 days between cities, considering the incubation period of SARS-CoV-2. Data collection was carried out in person at home, and the interviews lasted 30–45 min using a questionnaire in the DataGoal application via tablets. The questionnaire included questions on sociodemographic and economic variables, lifestyle habits, and general health status.

### 2.3. Habitual Consumption and Excessive Episodic of Alcohol

Two patterns of alcohol consumption were analyzed as outcome variables: habitual consumption of alcoholic beverages and excessive episodic alcohol consumption. All questions regarding alcohol consumption were based on questionnaires from the 2013 National Health Survey [30] and the 2019 Surveillance of Risk and Protective Factors for Chronic Diseases by Telephone Survey [7].

Regarding the habitual consumption of alcoholic beverages, the question was, “Currently, how often do you usually consume an alcoholic beverage?”. The responses were dichotomously grouped as follows: do not regularly consume alcoholic beverages when participants responded once or twice a month, three to four times a month, or did not use alcohol at all; habitual consumption was when participants responded that they consumed alcoholic beverages one to two times a week, three to four times a week, five to six times a week, or daily.

Excessive episodic consumption of alcohol, known as binge drinking, constitutes an excessive volume of alcohol in a short space of time and is characterized by five or more doses of standard drinks (one dose of alcoholic beverage or equivalent contains 12 g of pure alcohol, approximately 60 g) for men and four or more doses for women (48 g) in the last 30 days [1,8]. This variable was assessed using the question, “For men: in the last 30 days, have you consumed five or more doses of alcoholic beverages on a single occasion? For women: In the last 30 days, have you consumed four or more doses of alcoholic beverages on a single occasion?” and the answer options were no and yes.

### 2.4. Education and Sex

The explanatory variables included in the study were education level (<8 years, 9–11 years, and ≥12 years of study) and sex (female and male) [31].

Education was assessed through the following question: “To what grade and level did you study?” For a descriptive analysis of the sample, responses were grouped into the categories of less than eight years of study when the participants answered never having attended school, adult literacy, or incomplete and complete elementary school I (first to fourth grade) and II (fifth to eighth or ninth grade); between nine and eleven years of study when the participants answered incomplete and complete high school or incomplete higher education; and more than twelve years of study when participants responded having completed higher education, specialization, or postgraduate studies Latu Sensu, as well as postgraduate studies Stricto Sensu (master’s and/or doctorate).

For interaction analysis, responses were grouped dichotomously into the categories of less than nine years of study when participants responded having never attended school, adult literacy, or incomplete and complete elementary school I (first to fourth grade) and II (fifth to eighth or ninth grade), and greater than or equal to nine years of study when participants answered complete or incomplete high school, incomplete higher education, or completed higher education or specialization or postgraduate studies Latu Sensu, as well as postgraduate studies Stricto Sensu (master’s and/or doctorate).

### 2.5. Socioeconomic Characteristics

Socioeconomic characteristics consisted of age (18–34 years, 35–59 years, and ≥60 years), marital status (married and not married), children (no and yes), family income (≤2 minimum wages, >2 to ≤4 minimum wages, or >4 minimum wages) where the value of the minimum wage in force during 2020—the period of data collection—(BRL 1045.00) was considered, and skin color was self-reported (White or BBYI). Self-reported skin color was based on categories proposed by the Brazilian Institute of Geography and Statistics, which are White, Brown, Black, Yellow, and Indigenous. BBYI is an anacronym used in English for Black, Brown, Yellow, and Indigenous people. This evaluation was valid and consistent, as demonstrated by Travassos et al. [32].

### 2.6. Statistical Analysis

Statistical analyses were carried out considering the weighting factors of sampling and study design, using the package “svy” of software Stata^®^, version 15.1 (Stata Corporation, College Station, EUA). Categorical variables are described as relative frequencies and 95% confidence intervals (95%CI). The relationship between descriptive variables and outcomes was analyzed using Pearson’s chi-squared test.

Univariate and multivariate analyses were performed using logistic regression and odds ratios (OR), and their respective 95% confidence intervals were estimated. The models for habitual alcohol consumption and binge drinking were adjusted for age based on the literature [33,34].

An interaction analysis was performed to investigate the combined effect of education and sex on habitual consumption and excessive episodic of alcohol. The additive interaction evaluated two dichotomous exposures: education (A) and sex (B); that is, the interaction on an additive scale means that the combined effect of the two exposures was greater than the sum of the individual effects of the two exposures [35]. The interaction was analyzed using the logistic regression command following the dependent variable, variable A, variable B, and the adjustment variable. A significance level of 5% was used for all analyses. For the interaction analysis, the dichotomized education variable (<9 and ≥9 years of study) was used [31].

### 2.7. Ethical Aspects

Free and informed consent was obtained from all subjects involved in the study at the time of the interview, and all procedures were performed in accordance with the Brazilian guidelines and standards for research involving human beings in the Declaration of Helsinki. This study was approved by the Research Ethics Committee (Ethics Submission Certificate Nº. 32815620.0.1001.5149). This study adhered to the STROBE guidelines (Strengthening the Reporting of Observational Studies in Epidemiology).

## 3. Results

**Figure 1:**
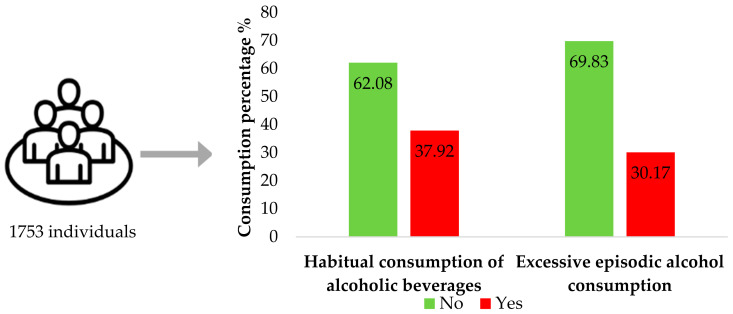
Prevalence of habitual alcohol consumption and excessive episodic alcohol consumption according to education and sex during the pandemic. COVID-Inconfidentes, 2020.

A total of 1753 individuals were evaluated. The prevalence of habitual alcohol consumption was 37.92%, and excessive episodic alcohol consumption was 30.17% (Figure 1). The majority were female (51.91%), aged between 35 and 59 years (45.52%), identified as non-White (74.33%), married (53.24%), and with children (74.07%). Furthermore, most participants reported having nine or more years of education (68.85%) and a family income of up to two times the minimum wage (40.97%; Table 1).

Habitual consumption was more prevalent among male participants (Table 1). Excessive episodic alcohol consumption was also more prevalent among male participants aged 35–59 years and among those with children (Table 2).

**Figure 2:**
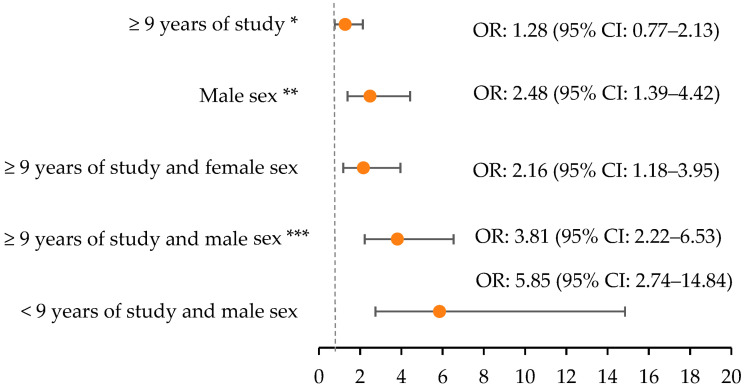
Multivariate logistic regression between years of study, sex, and habitual consumption of alcoholic beverages, 2020, COVID-Inconfidentes, Brazil (n = 1753). Note: CI, confidence interval; OR, odds ratio; age-adjusted analysis. * Reference category: <9 years of study; ** Reference category: female; *** Reference category: <9 years of study and female.

In Figure 2, it can be seen that in isolation, education is not associated with habitual alcohol consumption, whereas sex is associated, with males having 2.48 (95% CI: 1.39–4.42) times the chance of habitual alcohol consumption when compared to females. The interaction between education and sex stands out; it can be identified that participants with education greater than or equal to nine years and males (OR: 3.81; 95% CI: 2.22–6.53), education greater than or equal to nine years and female (OR: 2.16; 95% CI: 1.18–3.95), and less than nine years of education and male (OR: 5.85; 95% CI: 2. 74–14.84) were more likely to consume alcohol regularly than those with less than nine years of education and females.

**Figure 3:**
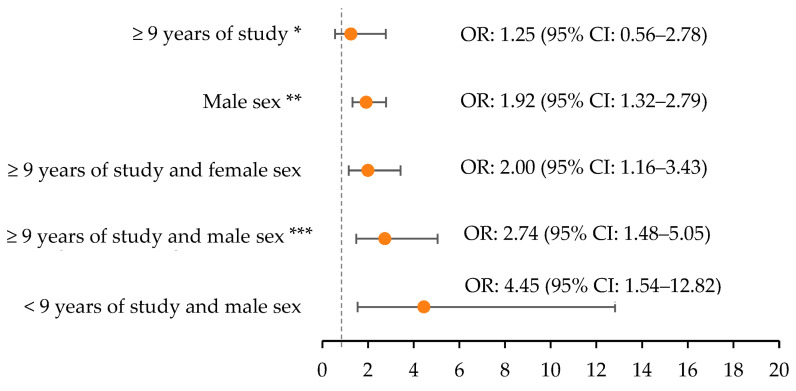
Multivariate logistic regression between years of study, sex, and excessive episodic alcohol consumption, 2020, COVID-Inconfidentes, Brazil (n = 1753). Note: CI, confidence interval; OR, odds ratio; age-adjusted analysis. * Reference category: <9 years of study; ** Reference category: female; *** Reference category: <9 years of study and female.

Regarding the pattern of excessive episodic alcohol consumption, it was observed separately that education is not associated with this pattern of consumption and that male individuals presented 1.92 (95% CI: 1.32–2.79) times the chance of reporting excessive episodic alcohol consumption when compared to females. However, the combination of education and sex increases the chances of this pattern of alcohol consumption; thus, individuals with education greater than or equal to nine years and males (OR: 2.74; 95% CI: 1.48–5.05); education greater than or equal to nine years and female (OR: 2.00; CI95%: 1.16–3.43); and education less than nine years and male (OR: 4.45; CI95%: 1.54–12.82) were more likely to have excessive episodic alcohol consumption when compared to individuals with less than nine years of education and females (Figure 3).

## 4. Discussion

To the best of our knowledge, this is the first study to investigate the joint association between education and sex in alcohol consumption during the period of social restrictions caused by the COVID-19 pandemic. The results showed a high prevalence of alcohol consumption by the study population during the pandemic and that education influenced alcohol consumption differently in men and women. Regarding the study hypothesis, the results suggest that the combination of education and sex increased the chances of habitual and episodic excessive alcohol consumption during the pandemic.

Our findings found that sex is a factor associated with habitual and episodic excessive alcohol consumption, with men being more likely to consume alcohol than women in both patterns. This is in line with the scientific literature, where recent results from a home health survey indicated that the percentage of alcohol consumption was higher among men than among women in the different consumption patterns investigated [8]. Excessive episodic alcohol consumption was estimated at 17.1%, with differences between sexes (men, 26%; women, 9.2%) [8]. Data from the World Health Organization also corroborate these findings regarding the disparity between sexes, showing that men consume more alcoholic beverages and are more involved in episodes of excessive consumption than women [1].

Therefore, differences in alcohol consumption between sexes have been widely explored in the literature, with consistent findings that males have a higher frequency and quantity of consumption than females, both globally and nationally [1,2]. In general, men are more likely than women to excessive episodic alcohol consumption in different societies [36]. Differences in the ethanol metabolization process and body composition partly explain the lower consumption of alcoholic beverages by women than men [37].

Furthermore, a review study discussed that differences in excessive episodic alcohol consumption by sex might be related to the social role of men and women in society, motivated by cultural factors, which may explain the greater consumption by men [38]. The wide variation in the magnitude of the male/female ratio observed in different contexts suggests that cultural influences, socioeconomic context, work, and the fact that alcohol is considered a symbol of masculinity contribute to the greater exposure of men to its consumption [39,40].

Although excessive episodic consumption was predominant among men, there was a greater tendency for this type of consumption to increase among women [8,41,42]. One hypothesis for this phenomenon is a change in women’s social roles and excessive workloads, which could lead to alcohol consumption as a mistaken form of self-medication for anxiety and stress [43]. Cultural prohibitions on alcoholic beverage consumption by women, children, and adolescents are common in most cultures. However, for those who break traditional prohibitions, vulnerability to the social consequences of alcohol consumption, particularly stigmatization, may increase, even becoming a barrier to treatment [41].

Furthermore, it is necessary to consider the synergism of socioeconomic inequalities in the health impacts observed in Brazil. Studies have shown that the pattern of alcohol consumption and its impact on health vary in the Brazilian population according to age, race/skin color, socioeconomic status, and/or education and that these factors may act differently between men and women [3,4,5].

Our results did not indicate an isolated association between education and alcohol consumption, which contradicts some studies in the literature where they found an association between education and alcohol consumption [14,44,45,46,47,48]. Brazilian surveys by Vigitel from 2019 prior to the pandemic and from 2020 and 2021 [7,49], as well as the data from the 2019 National Health Survey [7], demonstrated that excessive episodic alcohol consumption was higher in more educated individuals [50]. This indicates that high socioeconomic levels appear to be associated with a greater risk of alcohol consumption, which can be explained by the greater purchasing power of the population [12,51]. However, individuals with lower socioeconomic status are more susceptible to consequences related to alcohol dependence, with socioeconomic inequalities in mortality attributable to alcohol being greater than those from all causes [51].

When observing the combination of socioeconomic factors, the difference in the influence of education on alcohol consumption between the sexes found in the present study is notable. For females, having a high level of education increases the chance of drinking alcoholic beverages, while for men, there is no significant influence of education on alcohol consumption. These findings demonstrate that alcohol consumption among women is possibly related to purchasing power and opportunities, as higher levels of education generally result in better working conditions and higher income [52]. Among men, this is not strongly evident, given that alcohol consumption has always been high [6,8] and may have been accentuated as a result of psychological consequences in response to the stressful and anxiogenic situations experienced during the pandemic [21,22].

The intensification of alcohol consumption in circumstances of great financial and emotional instability and mourning situations has been previously documented in the literature, reinforcing the evidence that the occurrence of trauma or post-traumatic stress increases substance use disorder [53,54].

Furthermore, the data presented here encourage reflection on the reality of women during the pandemic, which refers to historical and cultural issues. With regards to working conditions, women’s vulnerability is manifested both in the greater probability of leaving the job market and becoming economically inactive than men, as well as in the overload of work in the domestic environment in the context of isolation. These make them more economically helpless or more prone to working in worse conditions [55,56]. This may also be related to fewer purchasing opportunities and is one of the possible explanations why women with less education are less likely to consume than women with more years of education.

Changes in socioeconomic conditions and lifestyles resulting from the COVID-19 pandemic provide a unique opportunity to explore the combined relationship between education and sex and how this affects alcohol consumption patterns. Although the results are paradoxical, the findings of the present study can be explained by the exacerbation of socioeconomic fragility resulting from the health crisis, as there is a greater risk of worse health behaviors in populations from lower socioeconomic strata [57].

Our findings confirm the hypothesis that education combined with sex (i.e., being male) would increase the consumption of alcoholic beverages during periods of social restrictions, given the inequalities in the impacts of the pandemic on the labor market [55,58] and the living conditions of the population [44]. Fear of becoming ill, financial uncertainty, and stress were common conditions during this period and were associated with increased alcohol use, depressive symptoms, increased stress, having children at home, and job loss [59,60,61]. The results showed that alcohol consumption could be reduced by interventions that consider the socioeconomic context and the most vulnerable groups.

In this study, the measurements were obtained through self-report and, therefore, were subject to memory bias. However, this research has several strengths, such as a robust sampling methodology with probabilistic selection and sample weights, which provides statistical power for the study. The interviews were conducted face-to-face, allowing greater precision in the information obtained. Furthermore, the data in the present study were derived from a population survey conducted during the pandemic, which is an important source of information on health situations and determinants.

The hypothesis addressed in this study was delimited and guided according to the current literature. Despite the methodological robustness, these findings must be interpreted with caution, for this study may not have national representatives, as they were conducted in only two municipalities in Brazil.

## 5. Conclusions

The high prevalence of alcohol consumption found in this study highlights a political, economic, social, and public health problem in the Brazilian population, which requires attention from government agencies and public policies. It is urgent to adopt strategies to reduce alcohol consumption; reinforce the importance of improvements in the implementation of labeling, pricing, and tax increase policies; support the intensification of inspection actions in relation to the sale of alcoholic beverages to minors aged <18 years; and adopt educational actions aimed at preventing and reducing alcohol consumption.

From a public health perspective, evaluating the modifying effect of sex on the relationship between education level and alcohol consumption allows us to expand our understanding of the combination of socioeconomic factors on health outcomes beyond traditional forms of analyzing these categories separately. Therefore, the need for future studies that evaluate the relationship between education and sex and alcohol consumption in other regions of Brazil is highlighted.

Furthermore, it is expected that this study will contribute to expanding the knowledge about the complex relationship between socioeconomic factors in determining the consumption of alcoholic beverages and will serve as support for proposing policies and actions aimed at reducing health inequalities.

## Figures and Tables

**Table 1:** Socioeconomic characteristics according to habitual consumption of alcoholic beverages, COVID-Inconfidentes, 2020 (n = 1753).

Characteristics	Total % (95%CI)	No % (95% CI)	Yes % (95% CI)	*p*-Value **
**Sex**				
Female	51.91 (44.73–59.01)	60.39 (54.37–66.11)	38.03 (26.2 8–51.37)	**0.002**
Male	48.09 (40.99–55.27)	39.61 (33.89–45.63)	61.97 (48.63–73.72)	
**Age**				
18–34 years	35.65 (31.19–40.37)	33.17 (28.17–38.58)	39.70 (30.85–49.29)	0.088
35–59 years	45.52 (41.00–50.12)	44.48 (38.51–50.60)	47.23 (38.47–56.17)	
>60 years	18.83 (15.49–22.70)	22.36 (18.56–26.67)	13.06 (08.58–19.39)	
**Skin color ^a^**				
White	25.67 (20.82–31.21)	26.41 (21.81–31.59)	24.46 (15.82–35.82)	0.722
BBYI	74.33 (68.79–79.18)	73.59 (68.41–78.19)	75.54 (64.18–84.18)	
**Marital status ^b^**				
Married	53.24 (47.19–59.19)	51.00 (45.68–56.31)	56.89 (44.37–68.59)	0.387
No married	46.76 (40.81–52.81)	49.00 (43.69–54.32)	43.11 (31.41–55.63)	
**Children**				
No	25.93 (22.00–30.29)	27.47 (23.09–32.33)	23.41 (16.85–31.55)	0.366
Yes	74.07 (69.71–78.00)	72.53 (67.67–76.91)	76.59 (68.45–83.15)	
**Education level**				
0–8 years	37.73 (32.52–43.25)	40.91 (34.92–47.18)	32.53 (24.01–42.40)	
9–11 years	41.47 (36.69–46.42)	39.85 (34.62–45.33)	44.13 (36.98–51.53)	0.206
≥12 years	20.79 (16.49–25.87)	19.24 (15.00–24.34)	23.34 (16.21–32.38)	
**Family income ^c^**				
≤2 MW	40.97 (35.46–46.72)	42.91 (36.41–49.67)	37.89 (30.12–46.33)	
>2 to ≤4 MW	32.03 (26.95–37.58)	32.97 (28.00–38.35)	30.55 (20.93–42.22)	0.264
>4 MW	27.00 (22.03–32.61)	24.12 (18.74–30.46)	31.56 (25.44–38.40)	

Ingesting alcoholic beverages at least once a week is considered habitual consumption, regardless of the amount of alcohol; CI: confidence interval; MW: minimum wage; ^a^ BBYI: Brown, Black, Yellow, and Indigenous; ^b^ not married: widowed, divorced, single; ^c^ value of the minimum wage at the time of data collection (2020): USD 1045,00 194.25 (1 USD = 5.3797 BRL); ** *p*-value for the chi-square test. Values in bold indicate statistical significance (*p* < 0.05).

**Table 2:** Socioeconomic characteristics according to excessive episodic alcohol consumption, COVID-Inconfidentes, 2020 (n = 1753).

Characteristics	No % (95% CI)	Yes % (95% CI)	*p*-Value **
**Sex**			
Female	56.81 (49.45–63.88)	40.57 (31.51–50.31)	**<0.001**
Male	43.19 (36.12–50.55)	59.43 (49.69–68.49)	
**Age**			
18–34 years	34.59 (27.51–42.43)	38.09 (29.12–47.97)	**0.019**
35–59 years	42.05 (35.93–48.43)	53.55 (42.25–64.49)	
>60 years	23.36 (19.23–28.05)	8.36 (05.45–12.63)	
**Skin color ^a^**			
White	26.49 (20.95–32.88)	23.79 (17.78–31.06)	0.477
BBYI	73.51 (67.12–79.05)	76.21 (68.94–82.22)	
**Marital status ^b^**			
Married	55.42 (49.32–61.36)	48.18 (37.47–59.07)	0.177
No married	44.58 (38.64–50.68)	51.82 (40.93–62.53)	
**Children**			
No	23.38 (19.73–27.47)	31.84 (23.70–41.26)	**0.044**
Yes	76.62 (72.53–80.27)	68.16 (58.74–76.30)	
**Education level**			
0–8 years	39.85 (33.18–46.93)	32.83 (21.14–47.12)	
9–11 years	40.87 (34.16–47.93)	42.88 (33.50–52.80)	0.453
≥12 years	19.28 (15.01–24.42)	24.29 (17.14–33.23)	
**Family income ^c^**			
≤2 MW	39.41 (32.74–46.50)	44.50 (32.23–57.47)	
>2 to ≤4 MW	32.08 (26.43–38.31)	31.93 (23.56–41.65)	0.602
>4 MW	28.51 (21.67–36.52)	23.58 (16.91–31.86)	

Excessive episodic alcohol consumption constitutes an excessive volume of alcohol in a short time; CI: confidence interval; MW: minimum wage; ^a^ BBYI: Brown, black, yellow, and indigenous; ^b^ Not married: widowed, divorced, single; ^c^ Value of the minimum wage at the time of data collection (2020): USD 1045,00 194.25 (1 USD = 5.3797 BRL); ** *p*-value for chi-square test. Values in bold indicate statistical significance (*p* < 0.05).

## Data Availability

The data presented in this study are available on request from the corresponding author. The data are not publicly available due to ethical reasons.
