# Correction: Diniz et al. The Interaction between Education and Sex with Alcohol Consumption during the COVID-19 Pandemic: A Cross-Sectional Analysis of Two Brazilian Cities. *Int. J. Environ. Res. Public Health* 2024, *21*, 804

**DOI:** 10.3390/ijerph21121627

**Published:** 2024-12-06

**Authors:** Amanda Popolino Diniz, Raquel de Deus Mendonça, George Luiz Lins Machado-Coelho, Adriana Lúcia Meireles

**Affiliations:** 1 Postgraduate Program in Health and Nutrition, Nutrition School, Federal University of Ouro Preto, Ouro Preto 35400000, MG, Brazil; amanda.diniz@aluno.ufop.edu.br; 2 Department of Clinical and Social Nutrition, School of Nutrition, Federal University of Ouro Preto, Ouro Preto 35400000, MG, Brazil; raquel.mendonca@ufop.edu.br; 3 Epidemiology Laboratory, Medical School, Federal University of Ouro Preto, Ouro Preto 35400000, MG, Brazil; gmcoelho@ufop.edu.br

There was an error in the original publication [1]. The variable of education was not clearly described as being categorized into three groups for the descriptive analysis of the sample and dichotomously for the analysis of the additive interaction between education, sex, and the dependent variables of the study.

A correction has been made to Materials and Methods, Education and Sex, Second Paragraph:

Education was assessed through the following question: “To what grade and level did you study?” For a descriptive analysis of the sample, responses were grouped into the categories of less than eight years of study when the participants answered never having attended school, adult literacy, or incomplete and complete elementary school I (first to fourth grade) and II (fifth to eighth or ninth grade); between nine and eleven years of study when the participants answered incomplete and complete high school or incomplete higher education; and more than twelve years of study when participants responded having completed higher education, specialization, or postgraduate studies Latu Sensu, as well as postgraduate studies Stricto Sensu (master’s and/or doctorate).

For interaction analysis, responses were grouped dichotomously into the categories of less than nine years of study when participants responded having never attended school, adult literacy, or incomplete and complete elementary school I (first to fourth grade) and II (fifth to eighth or ninth grade), and greater than or equal to nine years of study when participants answered complete or incomplete high school, incomplete higher education, or completed higher education or specialization or postgraduate studies Latu Sensu, as well as postgraduate studies Stricto Sensu (master’s and/or doctorate).

The authors state that the scientific conclusions are unaffected. This correction was approved by the Academic Editor. The original publication has also been updated.
